# Supplementary material for: Redox Regulation of Salt Tolerance in Eutrema salsugineum by Proteomics
Source: Int J Mol Sci. 2023 Sep 25;24(19):14518. doi: 10.3390/ijms241914518 (PMC10572166; doi:10.3390/ijms241914518)
Supplement: Supplementary file 1 [file ijms-24-14518-s001.zip › Supplement Figures.pdf]

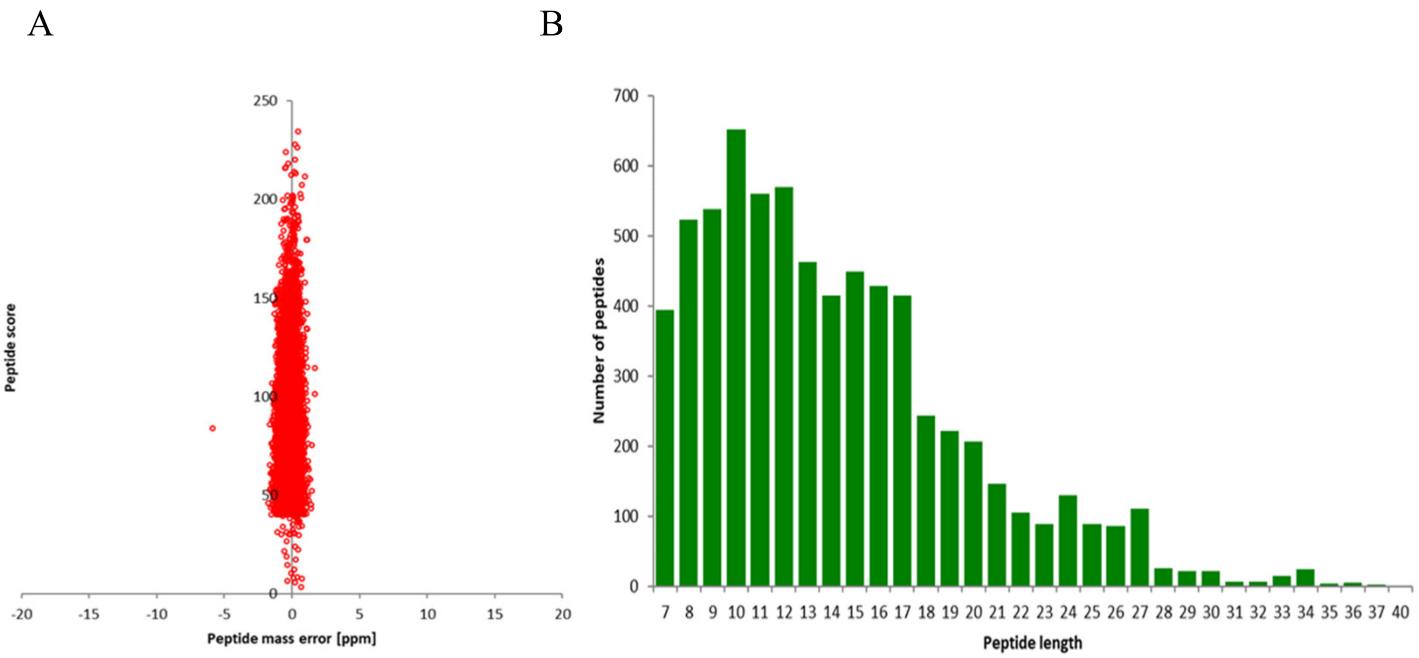

Figure S1. (A) Mass error of identified oxidized peptides. (B) Distribution of peptide lengths detected with oxidized sites by MS.

Raw file	Scan number	Mass analyzer	Score	m/z	Proteins
F9201TPRoB1_02	11736	FTMS	76.586	693.12	V4LRG4

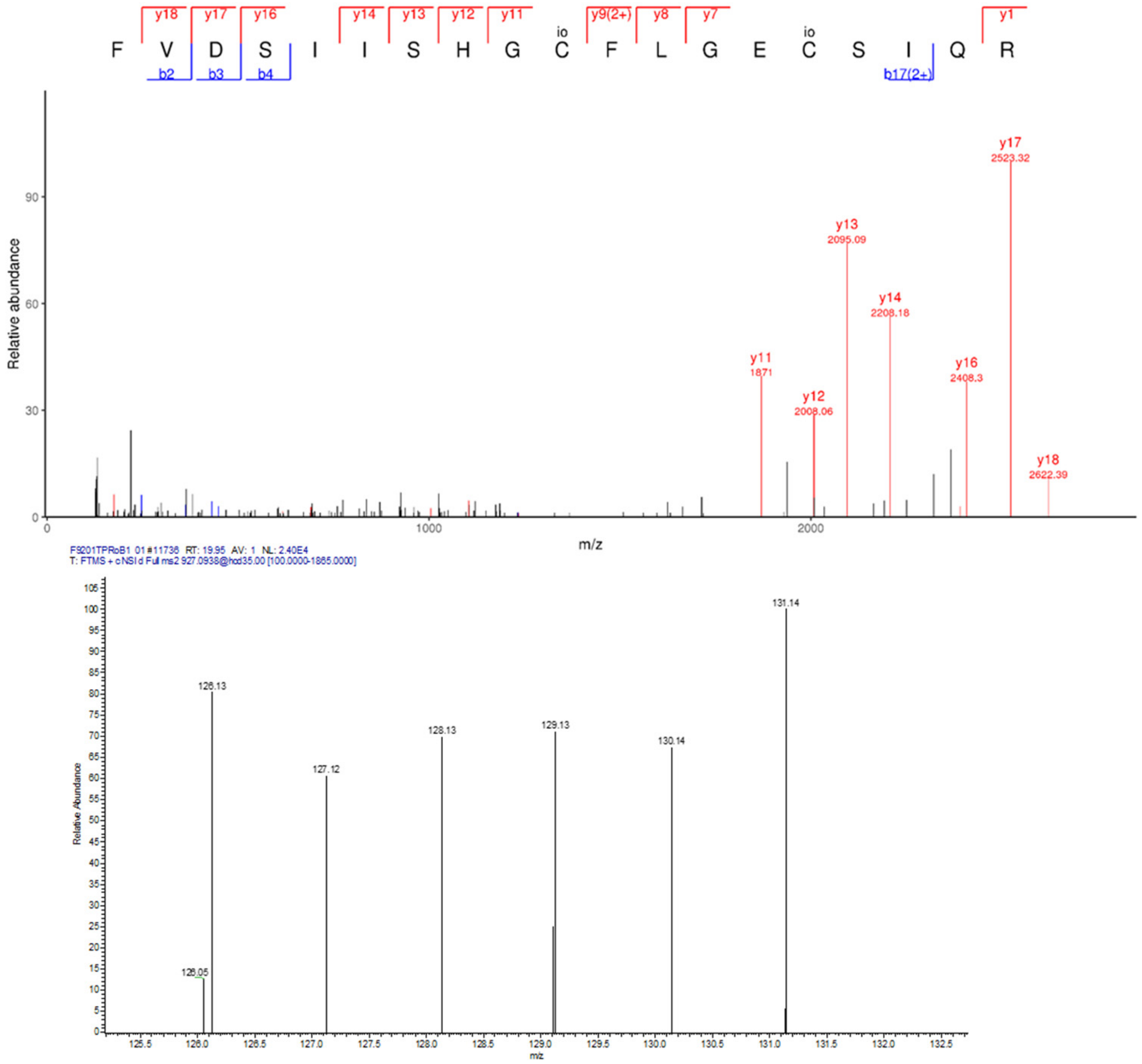

Figure S2. A representative MS/MS spectra of oxidative modified peptide identified in the redox proteome analysis from *Eutrema salsugineum*.
