# Supplementary material for: Redox Regulation of Salt Tolerance in Eutrema salsugineum by Proteomics
Source: Int J Mol Sci. 2023 Sep 25;24(19):14518. doi: 10.3390/ijms241914518 (PMC10572166; doi:10.3390/ijms241914518)
Supplement: Supplementary file 1 [file ijms-24-14518-s001.zip › Table S1.pdf]

Table S1. Significantly changed redox proteins in response to NaCl treatment in *Eutrema salsugineum*.

Protein Accession	Protein Description	Peptide	Redox TMT FC	TMT FC
EUTSA_v10004581mg	Peroxidase	MHFHDC^{76}FVQGC^{81}DGSVLISGTNTER	0.77	0.0261
EUTSA_v10004595mg	Nitrile-specifier protein 5 isoform X2	WVEYPAAGEAC^{171}K	0.485	0.116
EUTSA_v10023425mg	Monodehydroascorbate reductase	YGSLIIATGC^{174}TASR	1.314	0.088
EUTSA_v10011486mg	Chorismate synthase	GNSVGGVVTC^{264}IVR	1.246	0.036
EUTSA_v10018565mg	Epidermis-specific secreted glycoprotein EP1-like	C^{398}LGFFYNR	1.238	0.589
EUTSA_v10019820mg	Glutamate decarboxylase	C^{100}VNIIAR	0.777	0.081
EUTSA_v10003678mg	Beta-xylosidase 4	DVC^{394}TPANQELAADAAR	0.69	0.164
EUTSA_v10004123mg	Stress up-regulated Nod 19 protein	SDFILVNNGGLC^{142}R	0.382	0.649
EUTSA_v10005229mg	Defensin-like protein 2	C^{50}LSETNC^{56}K	2.77	0.124
EUTSA_v10013708mg	Regulatory particle triple-A ATPase 3	LVFQVC^{334}TSK	1.552	0.242
EUTSA_v10007958mg	mRNA, clone: RTFL01-15-D07	SVDDVQLC^{112}YDHLAFR	0.76	0.215
EUTSA_v10010563mg	Alpha/beta-Hydrolases superfamily protein	EIVVLC^{90}HGFR	0.753	0.096
EUTSA_v10014742mg	Plasmodesmata callose-binding protein 2	TLDYAC^{41}GNGADC^{47}NPTHPK	0.751	0.074
EUTSA_v10021555mg	Glycoprotein membrane precursor GPI-anchored	NENAEC^{57}LADEIADQFK	0.555	0.047
EUTSA_v10016929mg	ATP synthase subunit gamma	GLC^{117}GGINSTVVK	1.274	0.094
EUTSA_v10012883mg	GTP-binding protein TypA/BipA homolog	VC^{322} TSEDSC^{123}R	0.712	0.289
EUTSA_v10013803mg	Formate dehydrogenase, mitochondrial	C^{253} DVVVVNTPLTEK	2.304	0.139
EUTSA_v10010122mg	Alpha-1,4 glucan phosphorylase	AQQIC^{281}TVLYPGDATESGK	1.691	0.106
EUTSA_v10010363mg	O-Glycosyl hydrolases family 17 protein	NDGSC^{436}NFSGTGVVVGNNPSNGAC^{454}K	0.626	0.238
EUTSA_v10024927mg	Glucose-1-phosphate adenylyltransferase	FVDSIISHGC^{412}FLGEC^{417}SIQR	1.441	0.294

EUTSA_v10021123mg	Peroxidase	MHFHDC^{63}FVR	0.529	0.144
EUTSA_v10025296mg	Cystine lyase CORI3-like	LTADDVFMTVGC^{109}K	0.773	0.034
EUTSA_v10027346mg	Glutathione S-transferase family protein	YIC^{226}GNTLTEADIR	2.1	0.163
EUTSA_v10021132mg	Polygalacturonase inhibitor 1-like	TDC^{63}C^{64}TSWTGVEC^{72}TNSR	1.238	0.153
EUTSA_v10026076mg	Expansin-like B1	GAC^{48}GYGEFGR	3.705	0.19
EUTSA_v10021128mg	Guanine nucleotide-binding family protein	LWNTLGEC^{138}K	1.595	0.051
EUTSA_v10001940mg	Malic enzyme	C^{567}AESSMYSPTYR	0.606	0.087
EUTSA_v10021592mg	Putative gamma-glutamylcyclotransferase	TTEPSSS C^{128} VVEAYYAHK	0.509	0.074
EUTSA_v10017319mg	Glutathione peroxidase	C^{41}GLTDANYK	1.562	0.051
EUTSA_v10019955mg	ATPREP1	C^{621}VPSLNLGDIPK	0.819	0.089
EUTSA_v10025494mg	Cysteine protease RD19A-like	NIC^{355}GVDSLVSTVSATVSTTAH	0.609	0.061
EUTSA_v10020583mg	Galactokinase	ELAQLTC^{198}DC^{200}ER	1.615	0.233
EUTSA_v10024387mg	Beta-galactosidase	FASFGTPLGTC^{791}GSYQQGDC^{799}HAATSYAILER	2.089	0.059
EUTSA_v10024796mg	Pectin acetylesterase	NAQNAILSGC^{170}SAGALAAILHC^{181}DTFR	1.86	0.118
EUTSA_10017148mg	BnaA05g11250D protein	GC^{134}EVIVSGK	1.438	0.125
EUTSA_v10017125mg	Aconitase C-terminal domain-containing protein	EHAPVC^{158}LGAAGAK	1.481	0.145
EUTSA_v10026741mg	40S ribosomal protein S29-like	YGLNC^{38}C^{39}R	1.887	0.08
EUTSA_v10026489mg	BnaA01g13350D protein	VYDVIYQFIPKSEDTC^{112}AC^{114}K	1.345	0.075
EUTSA_v10007845mg	S-adenosylmethionine synthase	AIGFVSDDVGLDADNC^{90}K	1.497	0.091
EUTSA_v10002592mg	GDSL esterase/lipase At2g04570	NNPFTC^{317}TNADK	0.732	0.038
EUTSA_v10013021mg	Low-temperature-induced 65 kDa protein-like	C^{277}GEGFPAGFGGESGAGVGK	0.601	0.007
EUTSA_v10021148mg	Glutamyl-tRNA reductase-binding protein	TPQC^{139}TIQGSIGRPEDDNVR	1.24	0.083
EUTSA_v10021099mg	3-hydroxyacyl-CoA dehydrogenase family protein	TTVC^{219}SQDYAGFIVNR	1.965	0.076

EUTSA_v10017239mg	40S ribosomal protein S5-1-like	AQC[69]PIVER	1.319	0.144

The sequence of redox sensitive peptide was identified by iodoTMT method. The number in the upper right corner of Cys site was the position of this site in the amino acid sequence. Red or blue font color represent a significant increase or decrease in the abundance of the protein under the corresponding NaCl treatment conditions. Redox TMT FC represents leaf NaCl response to redox sensitive polypeptides. TMT FC represents corresponding protein abundance. FC values were presented as mean.
